# Naturally acquired antibodies to polymorphic and conserved epitopes of *Plasmodium falciparum* merozoite surface protein 3

**DOI:** 10.1111/j.1365-3024.2007.00951.x

**Published:** 2007-08

**Authors:** F H A OSIER, S D POLLEY, T MWANGI, B LOWE, D J CONWAY, K MARSH

**Affiliations:** 1 London School of Hygiene and Tropical Medicine Keppel Street, London, WC1E 7HT, UK; 2 KEMRI/Centre for Geographic Medicine Research-Coast Kilifi, Kenya; 3 Medical Research Council Laboratories, Fajara The Gambia, West Africa

**Keywords:** *allele-specific*, *antibodies*, *immunity*, *MSP3*, Plasmodium falciparum

## Abstract

*Many studies on the role of merozoite surface protein 3 (MSP3) in immunity against malaria have focused on a conserved section of MSP3. New evidence suggests that polymorphic sequences within MSP3 are under immune selection. We report a detailed analysis of naturally-acquired antibodies to allele-specific and conserved parts of MSP3 in a Kenyan cohort. Indirect and competition ELISA to heterologous recombinant MSP3 proteins were used for antibody assays, and parasites were genotyped for msp3 alleles. Antibody reactivity to allele-specific and conserved epitopes of MSP3 was heterogenous between individuals. Overall, the prevalence of allele-specific antibody reactivity was significantly higher (3D7-specific 54%, K1-specific 41%) than that to a recombinant protein representing a conserved portion of C-terminal MSP3 (24%,* P < *0·01). The most abundant IgG subclass was IgG3, followed by IgG1. Allele-specific reactivity to the K1-type of MSP3 was associated with a lower risk of clinical malaria episodes during a 6-month follow-up in individuals who were parasitized at the start of the malaria transmission season (Relative risk 0·41 with 95% confidence interval 0·20–0·81,* P = *0·011). The potential importance of allele-specific immunity to MSP3 should be considered in addition to immunity to conserved epitopes, in the development of an MSP3 malaria vaccine.*

## INTRODUCTION

Recent estimates of the global burden of malaria indicate that about 515 million clinical attacks were attributable to *Plasmodium falciparum*in 2002, with the majority (70%) of these episodes occurring in Africa (1) where young children account for 90% of all malaria deaths (2). An effective, safe and affordable malaria vaccine would be of enormous benefit for resource-poor countries, but remains elusive (3). Understanding the naturally acquired immunity (NAI) to malaria that is evident in older children and adults living in endemic areas is important for the selection of realistic vaccine targets and provides a reference against which the effectiveness of new vaccines can be compared. Although the mechanisms underlying NAI are incompletely understood, there is strong evidence to suggest that antibodies are centrally important (4,5). Red cell invasion is a critical point in the life cycle of parasite at which merozoites are briefly exposed to the host immune system and can therefore be targeted by antibodies. Naturally occurring antibodies to many merozoite recombinant antigens have been demonstrated in malaria endemic populations. Some of these antigens are currently under development as components of experimental vaccines.

Merozoite surface protein 3 (MSP3) is a polymorphic parasite antigen that may have a role in parasite invasion, as truncation of the *MSP3*gene reduces parasite invasion of erythrocytes (6). Structurally, MSP3 is composed of three blocks of four heptad repeats with the motif AXXAXXX, a hydrophilic region and a putative leucine zipper sequence at the C-terminus (7). Variation between alleles of *MSP3* in *P. falciparum*is due to substitutions and deletions in nonrepetitive sequences within and flanking the region encoding the alanine-heptad repeat domains. Comparison of sequences of *P. falciparum*from isolates collected from diverse geographical locations reveals a distinct dimorphism, with allelic sequences falling into major types, *3D7-like* and *K1-like* (8).

MSP3 is currently under development as a malaria vaccine candidate, and evidence that it is a target of NAI is accumulating. A vaccine incorporating a portion of the conserved C-terminal of MSP3 was safe, immunogenic and induced antibodies with a strong anti-parasite effect (9), which was still evident 12 months post-vaccination (10). Antibodies to this conserved portion of MSP3 mediate antibody dependent cellular inhibition (ADCI) of parasite growth in conjunction with monocytes *in vitro* (11), and in an immunodeficient *in vivo* mouse model (12,13). In vaccination experiments with MSP3, *Saimiri sciureus* (14) and *Aotus nancymai* (15) monkeys have been protected from lethal challenge with malaria. More recently, in longitudinal studies in endemic populations, antibodies to both conserved (13,16,17) and allele-specific (18) epitopes of MSP3 have been associated with reduced risk of malaria. Finally, population genetic analyses indicate that MSP3 may be under selection that maintains the polymorphisms (18).

We investigated the prevalence of allele-specific and conserved antibodies to *Escherichia coli*expressed recombinant MSP3 proteins, and determined whether these antibodies were associated with protection in a longitudinal study in a rural community in Kenya. We also examined the subclass pattern of IgG antibodies to MSP3 and the allele frequencies of *msp3* in the local parasite population.

## MATERIALS AND METHODS

### Study population

The study was conducted in Chonyi, a rural village in Kilifi district on the Kenyan Coast, which typically experiences two seasonal peaks in malaria transmission (June to August, November to December). The average annual entomological inoculation rate (EIR) is between 20 and 100 infective bites/person/year (19). Informed consent was obtained from all study participants and epidemiological details of this longitudinal cohort are published (20). Briefly, in October 2000, a cross-sectional survey was conducted in which venous blood samples were obtained for thick and thin peripheral blood smears to detect malaria parasites, and the separated serum was stored for assays of anti-malarial antibodies. In the ensuing 26 weeks, study participants were followed up for clinical episodes of malaria by both active and passive case detection. Episodes of malaria were monitored by weekly visits to the participant's homes, where temperatures were recorded and the presence of parasitaemia detected by microscopy of Giemsa-stained thick and thin peripheral blood smears, on a single slide that was prepared for those found to be febrile. One hundred high power fields were examined before a slide was reported as negative. Participants found to be unwell were treated and had open access to an outpatient clinic at the local district hospital. Malaria was defined as a fever of greater than 37·5°C with a parasitaemia of greater than 2500/µL of blood in subjects over a year old. For infants (< 1 year-old) a fever (axillary temperature over 37·5°C) plus any parasitaemia was counted as a clinical episode of malaria. These have been determined to be the optimal definitions for malaria in the different age-groups in this community (20). Data on 536 individuals were available for a minimum of 23 of the 26 weekly surveys, as previously described (21,22) and sera from these individuals are analysed here. Ethical approval was granted by the Kenya National Research Ethics Committee.

### Recombinant antigens and antibody assays

Full length recombinant antigens representing both of the dimorphic types of MSP3 (3D7 and K1), were used as previously described (18). Both antigens were expressed in *E. coli*as fusion products bound to maltose binding protein (MBP). At the amino acid level the two antigens differ both as a consequence of replacement polymorphisms (46 positions), as well as deletions, found in one sequence but not in the other (35 and 2 amino acid deletions in 3D7 and K1, with respect to each other), with the majority of the differences occurring in the N-terminal half of the sequence. A third antigen, representing a portion of the relatively conserved C-terminal of MSP3 (codons 234–354 of the 3D7 allele) was also expressed (18).

### Indirect ELISA

To measure specific IgG, indirect ELISA assays were performed in duplicate for each serum sample against each of the MSP3 proteins (3D7, K1 and C-terminal), and the fusion partner alone (MBP) using methods previously described (18). Briefly, individual wells of Dynex Immunolon 4HBX ELISA plates (Dynex Technologies Inc, Chantilli, VA, USA) were coated with 50 ng of antigen per 100 µL of carbonate coating buffer (15 mm Na_2_CO_3_, 35 mm NAHCO3, pH 9·3). *Plasmodium falciparum* (A4 strain) schizont extract was coated onto wells in PBS. Plates were incubated overnight at 4°C, after which wells were washed four times in PBS/Tween (Phosphate Buffered Saline/0·05% Tween 20), and blocked for 5 h at room temperature with 1% skimmed milk in PBS/Tween (blocking buffer). Wells were washed again and incubated overnight at 4°C with 100 µL of test sera (1/1000 dilution in blocking buffer). Plates were then washed four times and incubated for 3 h at room temperature with 100 µL of HRP-conjugated rabbit anti-human IgG (Dako Ltd, Buckinghamshire, UK) at 1/5000 dilution in blocking buffer before final washing and detection with H_2_O_2_ and *O*-phenylenediamine (Sigma, St. Louis, MO, USA). The reaction was stopped with 25 µL of 2 m H_2_SO_4_ per well and absorbance read at 492 nm. The optical density (OD) for antibodies to MBP alone was subtracted from that of antibodies to the K1, 3D7 and C-terminal MSP3. The cut-off OD for positivity was determined by taking the mean + 3 SD of 20 negative control sera from UK residents and was 0·26 for 3D7 MSP3, 0·32 for K1 MSP3 and 0·20 for C-terminal MSP3.

Competition ELISA assays were performed on samples that were positive for both the 3D7 and K1 MSP3 proteins (*n* = 258) to test for the presence of antibodies that distinguish each of the allelic types. For this assay, test sera were preincubated for 5 h with an excess of competing antigen (1000 ng) before following the indirect ELISA protocol for bound antigen. Any epitopes that are shared between the competing and plate bound antigens alleles would thus be blocked in the preincubation step. To confirm the presence of allele-specific reactivity, selected sera (*n* = 20) were further explored using competition ELISA with titred amounts of competing antigen (ranging from 0 to 1000 ng). Four sets of sera, each containing five samples, were selected as follows (1): sera with strong reactivity (OD > 2·0) to K1 MSP3 and very low or no reactivity to 3D7 MSP3 (2); sera with moderate/strong reactivity (OD > 1·5) to 3D7 MSP3 and very low or no reactivity to K1 MSP3 (3); sera that had strong reactivity (OD > 2·0) to both allelic antigens (4); sera that had moderate reactivity (OD 1·5–2·0) to both allelic antigens. Competing antigens were either homologous to plate coated antigen (e.g. K1 MSP3 coated on plate and K1 MSP3 preincubated with test sera) or heterologous (e.g. K1 MSP3 coated on plate and 3D7 MSP3 preincubated with test sera). For each sample, a difference in OD of > 0·3 between the heterologous and homologous assays was counted as evidence of allele-specific reactivity as this cut-off was considered to represent a substantial reactivity and gave consistent assessments of allele-specific reactivity when the assays were repeated on three separate days.

To determine which IgG subclasses against MSP3 were predominant, a subset of 96 sera that was positive for both MSP3 antigens were tested. The assays were identical to that of total IgG except HRP-conjugated polyclonal sheep antibodies specific for human IgG1, IgG2, IgG3 and IgG4 (The Binding Site, Birmingham, UK) at 1/3000 dilution were used as secondary antibodies. To estimate the amount of each of the IgG subclass antibodies bound to the antigens, standard curves were generated using titred amounts (doubling dilutions from 10 to 0·005 µg/mL) of purified human myeloma proteins coated on ELISA plates and used for interpolation of antibody amounts in test sera.

### Parasite *msp3* genotyping

Parasite DNA was available from blood samples collected at the cross-sectional time point in October 2000 from 85 individuals who were parasite positive, and was extracted from 100 µL of packed blood cells using the Qiamp DNA mini kit (Qiagen, UK). Allelic typing of the major dimorphism in *msp3* was performed by semi-nested PCR on genomic DNA followed by sequence-specific oligonucleotide probing (SSOP). The outer PCR primers were *370F* 5′-TGTACAGCTGCTTCAAAGG-3′ and *586R* 5′-CTCCTCCAAATTCCCAACC-3′, followed by a second round reaction using the same forward primer and *520R* 5′-TTGGTTTGCTTTTTGATAAGC-3′ as the reverse primer (primers designed to conserved sequences in *msp3*). PCR reactions were performed in 20 µL volumes in 96 well plates using 100 nm primers with initial template denaturation at 94°C for 2 min, followed by 44 cycles of 94°C for 1 min, 50°C for 1 min,and 72°C for 1·5 min, with a final extension of 72°C for 5 min at the end of the PCR. To discriminate the two major allelic types, SSOP was subsequently performed as previously described, with minor modifications (23,24). In brief, amplification products were denatured and 1·5 µL aliquots dotted onto duplicate nylon membranes (BioBond^TM^ Plus, Sigma). The membranes were then incubated with preheated (53°C) 3′ digoxigenin-labelled probes (MSP3-3D7, 5′-GCAAAAGATGATGCTGA-3′ and MSP3-K1, 5′-GCAAAGAAAGCTGCTGA-3′) in TMAC hybridization solution for 90 min at 53°C, with constant rotation in a hybridization oven, followed by high stringency washes in TMAC. The probes were detected using an alkaline phosphatase conjugated anti-digoxigenin antibody Fab fragment (Boehringer, Mannheim, Germany) and visualized using enhanced chemofluorescence (ECF) substrate (Amersham Phamarcia Biotech). Membranes were scanned on a Phosphorimager (STORM, Molecular Dynamics) and scored independently by two investigators. Most samples contained a single dominant allele (i.e. one allele was clearly much stronger than the other). Each allele was scored in samples that were strongly positive for both alleles.

### Statistical analysis

Data were analysed using stataversion 9·2 (Statcorp, TX, USA). The association between allele-specific antibody-positivity (3D7 MSP3 and K1 MSP3) or antibody positivity to the C-terminal of MSP3 in October 2000 and the proportion of individuals developing malaria during the 6-month follow-up period was investigated initially by univariate analysis, and subsequently by multivariate analysis that adjusted for age and reactivity to *P. falciparum* schizont extract using generalized linear models (GLM) for the binomial family that estimate the risk ratio (relative risk). Previous studies have shown that the presence of parasitaemia before the malaria transmission season (in October 2000) significantly correlates with outcome (increased risk of clinical malaria in the follow-up period) (21,22,25). Therefore the cohort was stratified into those who were slide positive or slide negative at the time of serum collection in October 2000 and then analysed for associations between antibodies and risk of subsequent disease in each stratum separately. Associations between the major allelic types of MSP3 (*3D7-like* or *K1-like*) in the infecting parasite, and the corresponding allele-specific reactivity were tested in samples that had a single dominant allele as previously described in a study of MSP2 (21). Antibody levels to 3D7 MSP3 were compared between individuals that had a dominant *3D7-like* allele and those with a dominant *K1-like* allele using the Wilcoxon rank sum test. Likewise, antibody levels to K1-MSP3 were compared between individuals that had a dominant *K1-like*allele and those that had a dominant *3D7-like* allele.

## RESULTS

### Heterogeneity of antibody reactivity to MSP3

**Figure 1:**
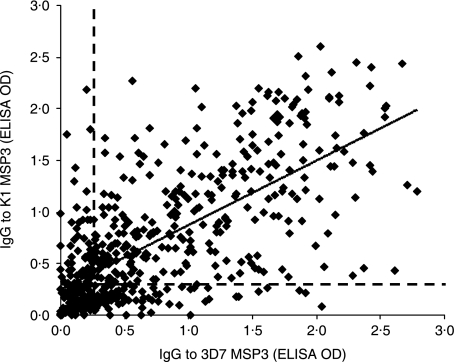
Correlation between antibody reactivity to K1 MSP3 and 3D7 MSP3 in the sera of 536 individuals from Chonyi village. Dashed lines indicate cut-off values for positive reactivity for 3D7 and K1 MSP3, respectively, as determined by mean + 3 SD of reactivity of 20 non-malaria exposed controls. Pair wise correlation coefficient was 0·6752, *P* = 0·0000, *n* = 536.

**Figure 2:**
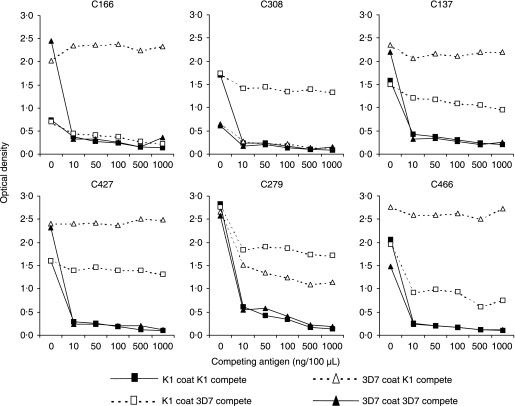
Competition ELISA using titred amounts of competing antigen on six representative sera. Data on remaining 14 sera are shown in supplementary figures.

There was a correlation between the reactivity of antibodies to the MSP3 full-length antigens 3D7 MSP3 and K1 MSP3 in the indirect ELISA, pair wise correlation coefficient 0·6752, *P* = 0·0000, *n* = 536 (Figure 1). However, it is clear from this scatter plot that many individuals only have strong reactivity to one of the full-length antigens and not to the other, while others have reactivity to both. In the competition ELISA using titred amounts of competing antigen in selected sera (*n* = 20), three different profiles of antibody reactivity were noted. The first profile comprised of sera that had allele-specific reactivity to epitopes on only one MSP3 antigen, and not the other (Figure 2; serum C166, 3D7-specific reactivity; serum C308, K1-specific reactivity). The second profile was of distinct mixed allele-specific reactivity to both antigens of MSP3 (Figure 2; serum C137 and C427). The third profile was reflected in sera that had both allele-specific, as well as conserved epitope reactivity (Figure 2; serum C279 and C466). None of the 20 sera tested in this fashion contained reactivity only to conserved epitopes.

### Predominance of allele-specific reactivity over reactivity to conserved epitopes

**Figure 3:**
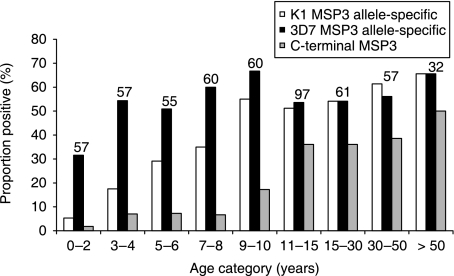
Age-specific prevalence of anti-MSP3 antibodies. Numbers above each group of bars indicate the number of individuals in each age category. The presence of allele-specific reactivity to 3D7 and K1 MSP3 was determined as follows: samples that were sero-positive (serum IgG OD level above the mean + 3 SD of that of 20 non-malaria exposed sera) for 3D7 MSP3 and sero-negative for K1 MSP3 were considered to be allele-specific for 3D7-MSP3, and likewise for K1 MSP3; samples that were sero-positive for both 3D7 and K1 MSP3 were tested by competition ELISA and differences of > 0·3 OD units between the heterologous and homologous competition assays were counted as evidence of allele-specific reactivity. Samples were considered to be positive for C-terminal MSP3 if the serum IgG OD level was above the mean + 3 SD of that of 20 non-malaria exposed sera.

One hundred and twenty-eight (24%) samples were sero-negative for both the full-length MSP3 antigens. Samples that were sero-positive for 3D7 MSP3 and not for K1 MSP3 in the indirect ELISA were considered to be allele-specific for 3D7 MSP3 (*n* = 109). Similarly, samples that were sero-positive for K1 MSP3 and not for 3D7 MSP3 were considered to be allele-specific for K1 MSP3 (*n* = 43). For samples that were sero-positive for both allelic antigens (*n* = 258), the presence of allele-specific reactivity was determined by competition ELISA (preincubation of test sera with 1000 ng of competing antigen). Of these 258 samples, 132 (51%) contained allele-specific antibodies to both 3D7 and K1 MSP3, while 29 (11%) contained antibodies to purely conserved epitopes. The remaining 97 (38%) samples contained antibodies to both allele-specific and conserved epitopes (allele-specific for 3D7 MSP3 and conserved, *n* = 50; allele-specific for K1 MSP3 and conserved, *n* = 47). As such, the overall prevalence of allele-specific reactivity was 54% (291/536) for 3D7 MSP3 and 41% (222/536) for K1 MSP3. The prevalence of reactivity to C-terminal MSP3 was 24% (126/531). The age specific prevalence of allele-specific reactivity (K1 and 3D7 MSP3) and reactivity to the C-terminal is shown in Figure 3. In the youngest children, allele-specific reactivity to 3D7 MSP3 was much more common than that of K1 MSP3, but its prevalence rose during the first 10 years of life so that in older children the prevalence of allele-specific reactivity was similar for both allelic antigens. The prevalence of reactivity to C-terminal MSP3 appeared to rise more slowly with age, being very low in children < 10 years old.

### IgG subclass reactivity to MSP3

**Figure 4:**
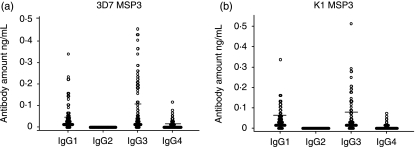
(a, b) IgG subclass reactivity to MSP3 recombinant antigens, (a) 3D7 MSP3 and (b) K1 MSP3, respectively. Horizontal bars indicate means (*n* = 96).

Selected sera (*n* = 96) that were sero-positive for antibodies to both K1 and 3D7 MSP3 were investigated for IgG subclass reactivity. For both MSP3 antigens, a predominance of IgG3 and IgG1 was found. A few subjects showed IgG4 reactivity, but virtually no reactivity was observed for IgG2 (Figure 4a,b).

### Allelic typing of *msp3*

A total of 85 parasite positive samples taken in October 2000 were genotyped for *3D7-like* and *K1-like* alleles of *MSP3*. Sixty samples (71%) had one dominant allele, either *3D7-like*(*n* = 52) or *K1-like*(*n* = 8). Twenty-five samples (29%) were strongly positive for the two alleles and both were counted. The total number of *3D7-like* and *K1-like* alleles counted was thus 77 and 33, with corresponding population allele frequencies of 0·7 and 0·3, respectively. The association between the infecting parasite genotype in a blood sample and the corresponding allele-specific antibody reactivity was analysed only in samples that contained one dominant allele. There was no difference in antibody levels (ELISA OD values) to 3D7 MSP3 in individuals that had parasites bearing a dominant 3D7-like allele compared to those bearing a dominant K1-like allele (Wilcoxon rank sum test *z* = –0·783, *P* = 0·434). Similarly, no difference in antibody levels to K1 MSP3 was observed in these two groups (*z* = 0·957, *P* = 0·339).

### Association between being positive for antibodies to MSP-3 alleles and protection from clinical malaria

The associations between being positive for antibodies to MSP3 in October 2000 and the risk of developing clinical malaria during the 6-month follow-up are presented in Table 1. The presence of allele-specific antibodies to K1 MSP3 was associated with a lower risk of clinical malaria in individuals who were parasitaemic in October 2000 before the start of the malaria transmission season. This association remained significant after adjusting for age and reactivity to parasite schizont extract in multivariate analyses. It also remained significant when antibody responses to AMA1 and MSP2 which have previously been shown to be associated with protection in this cohort (21,22) were taken into account (data not shown). Allele-specific antibodies to 3D7 MSP3 however, were not associated with a lower risk of malaria episodes. In a separate analysis, antibody levels to either of the full-length antigens (3D7 or K1 MSP3) were associated with a lower risk of malaria episodes (data not shown). Although antibodies to C-terminal MSP3 were associated with a lower risk of malaria in individuals who were aparasitaemic in October 2000 in the univariate analysis, this association was not significant when age and reactivity to schizont extract were taken into account.

**Table 1:** Antibodies to MSP3 in October 2000 and the risk of developing clinical episodes of malaria in the following 6 months

	Proportion^a^ acquiring malaria among individuals who were:		Univariate analysis		Multivariate analysis	
			
Antibody reactivity	IgG positive	IgG negative	Risk ratio (95% CI)	*P*-value	Risk ratio^b^ (95% CI)	*P*-value
All individuals (*n* = 536)						
Allele-specific K1 MSP3	8% (18/222)	20% (62/314)	0·41 (0·25–0·67)	0·000*	0·64 (0·38–1·08)	0·098
Allele-specific 3D7 MSP3	16% (46/291)	14% (34/245)	1·13 (0·75–1·71)	0·533	1·21 (0·81–1·79)	0·337
C-terminal MSP3^c^	8% (9/117)	17% (71/414)	0·44 (0·23–0·86)	0·018*	0·87 (0·44–1·69)	0·687
Slide positive individuals^d^ (*n* = 196)						
Allele-specific Kl MSP3	9% (9/101)	38% (36/95)	0·23 (0·11–0·46)	0·000*	0·41 (0·20–0·81)	0·011*
Allele-specific 3D7 MSP3	24% (28/119)	22% (17/77)	1·06 (0·62–1·81)	0·814	1·15 (0·83–1·60)	0·380
C-terminal MSP3^c^	16% (7/44)	25% (38/151)	0·63 (0·30–1·31)	0·220	1·37 (0·88–2·12)	0·158
Slide negative individuals^d^ (*n* = 340)						
Allele-specific K1 MSP3	7% (9/121)	12% (26/219)	0·62 (0·30–1·29)	0·206	1·14 (0·54–2·44)	0·717
Allele-specific 3D7 MSP3	10% (18/172)	10% (17/168)	1·03 (0·55–1·93)	0·916	1·07 (0·56–2·02)	0·824
C-terminal MSP3^c^	3% (2/73)	13% (33/263)	0·21 (0·05–0·88)	0·034*	0·39 (0·09–1·64)	0·202

Table shows the risk ratio (95% CI) of acquiring malaria among individuals who were positive for allele-specific antibodies to full-length MSP3 recombinant antigens or antibody positive to recombinant C-terminal MSP3 in October 2000 compared to those who were antibody negative.

a Number of individuals acquiring malaria/total number of individuals that were serum IgG positive or IgG negative.

b Risk ratio estimated from multivariate analyses adjusting for age and reactivity to *P. falciparum*schizont extract in generalized linear models.

c For reactivity to C-terminal MSP3 there were insufficient sera on five samples (slide all *n* = 531, slide positive *n* = 195, slide negative *n* = 336).

d Data stratified into two groups: individuals who were parasitaemic (or not) at the time of serum sampling in October 2000.

* *P* < 0·05.

## DISCUSSION

This study determined the prevalence of naturally acquired IgG antibody reactivity to MSP3 and investigated whether this correlated with protection from clinical episodes of malaria in a rural Kenyan community. Overall, allele-specific responses were more common than those to a recombinant antigen representing a conserved portion of MSP3 (54% to 3D7 MSP3 and 41% to K1 MSP3 vs. 24% to C-terminal MSP3). These results are similar to those of a study in The Gambia using similar recombinant MSP3 antigens (18). Of note, the recombinant C-terminal MSP3 used in this study does not include the sequence of a synthetic peptide (MSP3b) representing the conserved portion of the MSP3 sequence that was found to mediate ADCI (13,16,17). Antibodies to another merozoite surface protein MSP2 are also mainly directed against allele-specific rather than conserved epitopes (26–32). As with most malaria antigens, the prevalence of antibody to both the conserved and allele-specific epitopes of MSP3 increased significantly with age.

In the subset of individuals tested, a mixed IgG3 and IgG1 reactivity was observed. A similar IgG subclass distribution has been reported for reactivity to synthetic peptides representing conserved parts of MSP3, and IgG3 to conserved epitopes of MSP3 (MSP3b) has been associated with protection from malaria in field studies (13,17). Cytophilic antibodies to MSP3 have been shown to inhibit parasite growth in conjunction with monocytes both *in vivo* (11) and in passive transfer experiments in immunodeficient mice *in vitro* (12,13), in ADCI assays. Interestingly, vaccine induced antibodies to MSP3 were predominantly of the IgG1 subclass in malaria naive volunteers, while naturally acquired antibodies are mixed IgG3 and IgG1 (10). Nevertheless, vaccine-induced IgG1 to MSP3 was still present and capable of mediating ADCI of parasite growth *in vitro* 12 months after vaccination (10).

The *3D7-like* allele of *msp3* was three times more common in this population than the *K1-like* allele. This difference is reflected in the age prevalence of allele-specific reactivity with younger children acquiring antibodies to 3D7 MSP3 much earlier than those to K1 MSP3. Thus at population level, the prevalence of allele-specific antibody reactivity to MSP3 appears to reflect the prevailing allele-frequency of the local parasite in children who are rapidly acquiring natural immunity to malaria. At an individual level however, there was no difference in antibody levels between individuals who had either dominant allelic type of MSP3. Positive correlations between the infecting parasite genotype and the specific antibody response at the individual level have been observed for antibody responses to alleles of MSP1 block 2 in some (33,34) but not all studies (35,36).

Among individuals who were parasitaemic at the time of serum collection, allele-specific antibody responses to K1 MSP3 were associated with a significantly lower risk of malaria, even when the analyses were adjusted for age and reactivity to *P. falciparum* schizont extract. Allele-specific antibodies to 3D7 MSP3 and a C-terminal portion of MSP3 were not associated with a lower risk of malaria in this study. For antibodies to C-terminal MSP3, this was not surprising, given their low prevalence in this population. However, for allele-specific epitopes it was unexpected that antibodies targeted to the rarer allele would be associated with protection, while those directed against the commoner allele were not. It is not clear why this should be the case. In The Gambia, reactivity to both antigens was associated with a lower risk of malaria (18). More studies in areas of differing endemicity, and within different clinical categories of malaria will be necessary to build a picture of the protective effect of naturally acquired antibodies to MSP3. Nonetheless, this study contributes to the evidence that allele-specific antibodies to MSP3 are important in naturally acquired immunity to malaria and may add value to an MSP3 malaria vaccine.
